# How Could Private Healthcare Better Contribute to Healthcare Coverage in Vietnam?

**DOI:** 10.15171/ijhpm.2017.05

**Published:** 2017-01-22

**Authors:** Mai Phuong Nguyen, Andrew Wilson

**Affiliations:** ^1^Department of Medical Services Administration, Ministry of Health, Hanoi, Vietnam.; ^2^The Menzies Centre for Health Policy, University of Sydney, Sydney, Australia.

**Keywords:** Health System, Public Sector, Private Healthcare, Health Coverage, Vietnam

## Abstract

Private healthcare services in Vietnam are seen as a major part of the solution to the rapid increase in need and demand for healthcare services. Formally recognized over 20 years ago, the private health services coexist with public services and are available all over the country. However, the scale and size of private sector is still small compared to the public sector and public acceptance and reputation still limited. There are substantial concerns with the quality of services and the adequacy of regulation. Human resource for health is currently inadequate to support growth in both public and private sectors. The role of the private sector in achieving Vietnam’s population health objectives is not clear. In this emerging economy, there is significant potential for increased dependency on private healthcare to increase health access inequities. This paper discusses how private healthcare could better contribute to healthcare coverage in Vietnam.

## Introduction

The Vietnamese health system is now a mix of public and private services of which the former is dominant. During the early 1990s, economic and social reforms opened the door for private healthcare with the introduction of user charges and health insurance, liberalization of the pharmaceutical market, and legalization of private health activities.^1^ Along with subsequent economic growth, national health expenditure rose from 2.9% of the state budget in 2005 to 6% in 2013.^2^ The 2013 per capita health expenditure of US$111 was more than twice the level of 2006.^3^ However, in meeting the needs of a rapidly growing population with growing healthcare expectations, the public health system faces challenges of limited capacity or willingness to further increase public expenditure, constrained human resources for health, capacity and quality issues in service infrastructure.^4^ Increasingly the government is looking to private sector healthcare to address these challenges. This paper will discuss the possible contribution of private healthcare to the overall health system.

## Private Healthcare Services Distribution and Activity

Vietnam’s public health system is organized into four levels described as central, provincial, district, and communal. The main types of health establishments are hospitals, health centers, clinics and health stations, distributed throughout the country and staffed by 424 237 health workers.^5^ Private health facilities are also available at all levels as shown in Table. Most private hospitals are small scale and located in cities and towns, and yet comprise 4.2% of total hospital beds in Vietnam.^5^ Three quarter of these have less than 100 beds. There are a few large and better equipped private hospitals, which are staffed with highly skilled health professionals attracted from state health sector.^5,6^ A notable new development is that domestic corporations have started to create hospital and clinic chains across the country (Vingroup and Hoan My Corporations). The number of private clinics and other facilities is much greater than the public ones (Table).^5^ The private sector offers services for any persons willing and able to pay in both urban and rural areas.^7^ Indeed, people frequently seek care at a private health facility even though they live near public health facilities. The private sector offers a diverse range of services including pediatrics,^7,8^ elderly care,^9^ sexual and reproductive health,^10^ antenatal care,^11^ family planning^12^ as well as traditional medicines.

**Table:** Number and Distribution of Public and Private Health Facilities

**No.**	**Economic Regions**	**Hospitals**		**Clinics**		**Other Facilities**^a^		**Hospital Beds**	
		**Public**	**Private**	**Public**	**Private**	**Public**	**Private**	**Public**	**Private**
1	Red River Delta	242	37	83	116	11	4248	61284	1935
2	North Midlands and Mountain areas	167	4	153	20	14	1302	26 120	417
3	North and Central Coastal areas	205	32	101	58	12	3205	47 325	1678
4	Central Highlands	45	2	43	4	7	1161	8296	300
5	South East	104	57	42	296	16	4902	4316	4126
6	Mekong River Delta	147	23	60	22	28	6055	36 114	1045
	Total	910	155	482	516	88	20 873	222 855	9501

^a^Other facilities include those outside the hospitals and clinics as traditional medicine, para-clinical and health service units. All public commune health stations exclude in the table.

The number of non-state health workers accounts for 16.5% of total national health workforce.^5^ The human resources for the private sector are indirectly subsidized as all physicians and majority of other categories of health workers are graduates from public universities and colleges that are partially financed by the government. Moreover, many have dual positions in the public and private sectors.^13^

Private healthcare is paid for either by the patients directly or by their social health insurance (SHI) or private health insurance scheme. Private health expenditure comprises nearly 60% of total health expenditure and in 2013, 85% of that was direct payments (out-of-pocket expenditure).^3^

The governance characteristics of the private sector reflect its recent and unorganized development, its small business dominance and the immaturity of the regulatory and professional environment. The majority of services are owned by the health professionals working in them or by small private investor groups in the case of small or medium-sized medical facilities. In terms of ownership of private medical businesses, domestic investors can own any size business based on their financial capability. For foreign investors, according to Vietnam’s World Trade Organization commitments on services, they can invest 100% of the capital costs of medical businesses or cooperate under the form of joint venture and business cooperation contracts. However, the minimum investment is US$20 million for a hospital, US$2 million for a general clinics, and US$200 000 for specialized clinics.^13^

Professional associations can provide a quasi-regulatory role on healthcare providers and on their businesses. These associations are allowed by the government to gather, organize, and support its members, preserve and protect the reputation and honor of the physician and their professions in accordance with the law; to consult, judge and monitor on issues related to private health practice, recommend to the government to formulate policies suitable to private health activities; and to protect the legal rights of its members. However, the scope of such associations’ activities and their position has not been defined by law. At this stage in their development, they are little more than professional forums, taking little responsibility for monitoring quality. They also face difficulties with financial and facilities sustainability.

## Future Perspectives

### The Role of the Private Sector in Achieving Healthcare Coverage

Morgan and colleagues^14^ have identified the performance characteristics of a private sector to best support a mixed health system to achieve the universal health coverage. As an integral part of the overall Vietnamese healthcare system, the private sector should contribute to achieving three dimension of overall system performance; namely equity, quality, and efficiency.

Theoretically, the private sector could enhance equity by meeting the needs of those who can afford to pay allowing publicly funded services to be focused on those who can afford them. A major factor other than population growth impacting on demand for healthcare is the growing middle and affluent class (expected to be 33 million people by 2020^15^) especially in urban areas. Clients from middle and wealthy classes are willing to pay more to meet their individual requirements and have higher expectations of service. Therefore, a private sector with modern clinics and hospitals is a likely option to meet this demand. However, there is a risk that this approach may drive up the overall costs of healthcare, drain healthcare workers from the public sector and lead to two tiers in even essential services.

A better organized and regulated private sector could also help to improve access to quality care for the 60.7 million people (nearly 68% of total population) living in rural areas. State-owned commune health centers are frequently not truly geographically local and the private sector is already filling gaps in these services. However, these private services are of variable quality and currently poorly regulated.

The quality of private health services varies by type of services and local context. In general, the quality of private health services is usually poorer than public services, especially in rural areas.^16^ Moreover, the nature of the private sector at present, with large numbers of small scale and unorganized private facilities, means that a visible systematic improvement in quality is unlikely to be easy to achieve without government regulation and third party accreditation. If the policy intent is for private sector healthcare services to carry an even greater proportion of the future healthcare demand then it is critical that the concerns about variability in quality are addressed nationally and systematically.

Empirical evidence from Vietnam shows that non-state hospitals demonstrate more flexibility in use of their health workforce and in the adaptation of different funding opportunities. They use different combinations of full-time, part-time, and retired doctors in response to different circumstances.^6^ In general, non-state hospitals have shorter average length of stays and higher utilization of outpatients compared to inpatients than state hospitals. To maximize profits, private clinics and other health facilities are highly incentivized to manage their resources efficiently. However, in the current private environment, the efficient allocation and management of resources is not necessarily synonymous with quality of service. Moreover, the profit driver also incentivizes over-servicing. Non-state hospitals offer a much higher number of diagnostic tests, especially X-rays and computerized tomography (CT) scans than would be expected based on the number of patients treated.^17^ There is a need for further examination of the policy options to balance these two objectives.

### Overcoming Barriers to Development

For private healthcare to effectively and efficiently contribute to meeting Vietnam’s health needs a number of barriers must be addressed.

- Although the legal framework (The Vietnam Constitution of 1992 and the Law of Medical examination and treatment of 2009)^18^ exists, the legislative efforts made to regulate private provision lack a contextual understanding of the private sector. There are no incentives to engage private providers in meeting national objectives, and no guidelines for public-private partnerships. While the aim of government in promoting private health provision is clear, there is an absence of long-term planning and strategy to direct development. Consequently, private sector is developing in unorganized and a market-oriented way.
- Private investment is still small, medium in size and short-term.^5^ There are few Vietnamese sources for large scale investment of the size necessary to build and commence large and modern hospitals.
- There are significant constraints on human resource for health as existing education and training providers are focused on meeting the public sector requirements. Currently, the private sector is not attractive to highly skilled and experienced staff. In the short term, this may serve a policy advantage for government to encourage the private sector to assist with meeting national objectives by cooperation and collaboration to exchange high skilled and qualified experts. However, if the private healthcare sector is to expand and do so without robbing the public sector of highly trained clinical staff then the skilled health workforce pipeline will need to be expanded, and this may require the participation of private sector in medical and other clinician training.
- There is a lack of data and information on private health activities and no research to systematically understand the aspects of private sector, its interaction with public one, that is necessary to inform government policy and potentially government contracting with the private sector.
- Self-regulation is poorly understood as a concept and largely non-functional. This includes professional standards about practicing beyond the scope of allowed expertise; limiting the ordering of pathology and radiology to that required for the immediate management of the patient, misleading advertising of professional competence; lack of price lists for services and over-charging. These flaws discredit private providers as professional in the mind of the community.

### Facilitating Further Private Sector Development

There are four critical steps to facilitate further development of a private sector that could contribute to, rather than undermine, universal healthcare in Vietnam. First, Vietnam needs to review, complete, and implement the system of regulations and instruments for the private health activities to ensure legal and professional equality between private and state providers as well as to govern appropriately the quality, safety and public accountability of the private services. The private sector should have a place in the National Health Strategy and Long-Term Plan. Its role in self-financing and delivering a certain type of services should be explicitly stated. For example, the private sector could substantially add to delivery of most curative services outside essential services while the public sector is likely to remain the main provider of preventive and public health services. The role of professional associations should be strengthened by giving more power and incentives for them to participate in the process forming, evaluating and monitoring the regulations. By taking the role of policy advocacy and discussion, trust can be build to help ensure increased accountability in the public and private sectors. This should be complemented by an appropriate separate and independent state complaints investigation and prosecution authority capable of addressing more serious concerns of healthcare negligence or quality in both public and private sectors.

An appropriate investment policy is critical in order to scale up the private sector and attract strong domestic and foreign investors committed to long term investment. The strategies to achieve this might be different for different size facilities. For medium-size hospitals (100-200 beds) the government’s strategies should focus on promoting adequate financial capacity among domestic investors because this size is compatible with likely domestic capital and management capacity. For hospitals more than 200 beds, the government may encourage participation of the larger international and domestic hospital corporations. Ensuring regulatory compliance may be easier with a move to larger facilities run by established brand hospital corporations that have well-established quality control and improvement practices.

Third, the private health sector’s important role in rural areas must be recognized, incentivized but appropriately controlled. A substantial proportion of the Vietnamese population still lives in under-serviced rural and semi-rural areas including mountainous regions where the inequity in access to healthcare among the ethnic minority groups is definitively documented. New residential developments in regional locations or suburbs of big cities present opportunities for private hospitals due to the cheaper and larger land and hiring opportunities, health demands of expanding populations and lower setup costs. The investment incentives including tax and land rent relief and increasing the capital loan support should be designed to promote private providers to regions lacking public health coverage. District and provincial planning needs to ensure that there are complementary public healthcare services so the existing inequities are not increased.

Fourth, accurate and unbiased information on the private sector is crucial for any political and social intervention. Therefore, health information systems and statistical support must be upgraded and periodic public reporting of private sector activities should be mandatory and part of the national health reports. Furthermore, there is a need for research (by academic organizations or government agencies providing it includes independent experts) to better understand the healthcare demands, service gaps, and economics of different service model options, both public and private.

It is important to be aware of possible downsides of expanding the private healthcare sector. There are significant risks in relying on private healthcare to grow to meet growing demands, including potential negative impacts on the public sector and on equity in health access unless the right regulatory and financial structures are in place. Out-of-pocket expenses on top of the SHI reimbursements are already high and healthcare costs a significant contributor to poverty. Without the appropriate controls, it is to be expected that many private providers will increase profits by charging higher medical fees where possible and using more technical services than necessary. Moreover, due to workforce shortages, private sector will need to create an environment to attract high-skilled workers, which usually includes offering higher salaries. The net consequence of this could be a substantial increase in healthcare costs above that necessary to meet healthcare need and the poor are more vulnerable in a private healthcare environment. Therefore, regulation, quality control, dialogue and partnership with private sector, communication and public education about health-promoting behaviors, health services use, and information on private providers are essential tools for the government to mitigate the downside of expanding private sector.

Finally, the public sector has a critical role in the stewardship and overseeing of the whole health system. The capacity of government institutions is important to effective regulation and management of the health market. Therefore, there is a need to invest in the education and training of government officials to improve their understanding and capability to work with private sector.

## Conclusion

Vietnam has successfully taken many progressive steps to develop and harness the private sector to achieve healthcare coverage. Despite these initial achievements, the scale and size of private sector is still small compared to the public one. To meet its full potential, there will need to be a significant expansion in capacity, both capital and human resources while addressing significant issues of quality and safety of the services and community trust is still to be achieved. Government needs to more clearly articulate the role it sees for the private healthcare sector in relation to achieving healthcare coverage and introduce regulation, financial incentives and planning to achieve this.

## Acknowledgments

The views expressed in this paper are fully the responsibility of the authors and do not necessarily reflect the views of the organizations they work for.

## Ethical issues

Not applicable.

## Competing interests

Professor Wilson is the Director of the NHMRC Australian Prevention Partnership Centre, Ultimo, Australia which is partly funded by the HCF Health and Medical Research Foundation. We declare that we have no other conflicts of interest.

## Authors’ contributions

Both authors contributed equally to the writing of this paper.

## Authors’ affiliations

^1^Department of Medical Services Administration, Ministry of Health, Hanoi, Vietnam. ^2^The Menzies Centre for Health Policy, University of Sydney, Sydney, Australia.
